# An Iron Shield to Protect Epigallocatehin-3-Gallate from Degradation: Multifunctional Self-Assembled Iron Oxide Nanocarrier Enhances Protein Kinase CK2 Intracellular Targeting and Inhibition

**DOI:** 10.3390/pharmaceutics13081266

**Published:** 2021-08-16

**Authors:** Luca Fasolato, Massimiliano Magro, Giorgio Cozza, Ferruccio Sbarra, Simone Molinari, Enrico Novelli, Fabio Vianello, Andrea Venerando

**Affiliations:** 1 Department of Comparative Biomedicine and Food Science, Agripolis Campus, University of Padova, Viale dell’Università 16, 35020 Legnaro, Italy; luca.fasolato@unipd.it (L.F.); massimiliano.magro@unipd.it (M.M.); ferrucciosbarra@gmail.com (F.S.); enrico.novelli@unipd.it (E.N.); 2 Department of Molecular Medicine, University of Padova, Via Gabelli 63, 35121 Padova, Italy; giorgio.cozza@unipd.it; 3 Department of Geosciences, University of Padova, Via Gradenigo 6, 35131 Padova, Italy; simone.molinari@unipd.it

**Keywords:** epigallocatechin-3-gallate, nanocarrier, protein kinase CK2

## Abstract

Protein kinase CK2 is largely involved in cell proliferation and apoptosis and is generally recognized as an Achilles’ heel of cancer, being overexpressed in several malignancies. The beneficial effects of (−)-epigallocatechin-3-gallate (EGCG) in the prevention and treatment of several diseases, including cancer, have been widely reported. However, poor stability and limited bioavailability hinder the development of EGCG as an effective therapeutic agent. The combination of innovative nanomaterials and bioactive compounds into nanoparticle-based systems demonstrates the synergistic advantages of nanocomplexes as compared to the individual components. In the present study, we developed a self-assembled core-shell nanohybrid (SAMN@EGCG) combining EGCG and intrinsic dual-signal iron oxide nanoparticles (Surface Active Maghemite Nanoparticles). Interestingly, nano-immobilization on SAMNs protects EGCG from degradation, preventing its auto-oxidation. Most importantly, the nanohybrid was able to successfully deliver EGCG into cancer cells, displaying impressive protein kinase CK2 inhibition comparable to that obtained with the most specific CK2 inhibitor, CX-4945 (5.5 vs. 3 µM), thus promoting the phytochemical exploitation as a valuable alternative for cancer therapy. Finally, to assess the advantages offered by nano-immobilization, we tested SAMN@EGCG against *Pseudomonas aeruginosa*, a Gram-negative bacterium involved in severe lung infections. An improved antimicrobial effect with a drastic drop of MIC from 500 to 32.7 μM was shown.

## 1. Introduction

(−)-Epigallocatechin-3-gallate (EGCG), a flavonoid belonging to the phytochemical class of catechins, deserves particular attention due to its widely recognized health potential [1]. In addition to antioxidant and radical scavenging properties, which have been exploited in the prevention and treatment of cardiovascular [2] and neurodegenerative diseases [3], EGCG has been proposed as an anti-cancer and/or cancer-preventive agent due to its ability to inhibit in vitro tumor formation and growth [4]. This ability has been described through the activation/inhibition of many signaling pathways either by interacting directly with specific protein targets or by the generation of reactive oxygen species (ROS) that may in turn trigger apoptosis in cancer cells [4]. Cancer is a multifactorial disease, and malignant growth requires the concomitant occurrence of a substantial number of alterations in cell physiology. In this context, protein kinase CK2, a pleiotropic enzyme deeply involved in key signaling pathways for tumor growth, represents a valuable molecular target because, albeit not being a direct cause of tumor development, its overexpression/hyperactivity is required to set the environmental basis for neoplasia growth and progression [5]. Notably, EGCG has the potential to be an appealing CK2 inhibitor, displaying an IC_50_ in the nanomolar range in in vitro assays [6], but it lacks its efficacy on endogenous enzymes when tested in cell cultures. Indeed, one of the major challenges in the use of natural compounds as therapeutic agents is due to their in vivo instability, as well as their poor bioavailability and targeting [7]. EGCG is not exempt from such limitations, showing poor bioavailability due to several factors, and the loss of activity in cell cultures may account for the intrinsic EGCG instability due to the susceptibility of the hydroxyl groups to auto-oxidation and rapid degradation in cell culture media [8]. In addition, metabolic transformations (i.e., methylation, glucuronidation, and sulfation) as well as active efflux by multidrug resistance-associated proteins further affect its efficacy in vivo [8]. It should be noted that pharmacokinetic studies of EGCG have been conducted mainly on green tea catechin extracts following oral administration [9,10]. In any case, it appears clear that sustained release by pharmaceutical formulations, which protect catechins from degradation/metabolism, is mandatory to enable the therapeutic use of such bioactive molecules [9]. Interestingly, the combination of innovative nanomaterials and bioactive compounds in nanoparticle-based systems demonstrates the synergistic advantages of nanocomplexes as compared to the individual components. Herein, we developed a self-assembled nanohybrid of EGCG and Surface Active Maghemite Nanoparticles (SAMNs) in order to improve the chemical stability and cell targeting of the active polyphenol compound. Among iron oxide nanoparticles, SAMNs offer several advantages and promising alternatives in the development of smart vehicles for drug delivery due to their peculiar physical and chemical features [11]. In addition to excellent colloidal stability in aqueous solution without any surface modification or coating derivatization, SAMNs allow the easy formation of stable core-shell complexes with selected molecules. Notably, bioactive molecules nano-complexed with SAMNs display enhanced activity and targeting in comparison to free soluble counterparts [11,12]. Interestingly, nano-immobilization of EGCG, exploiting the chelation of EGCG aromatic hydroxyl groups by under-coordinated Fe(III) on the SAMN surface, protect them from auto-oxidation, as confirmed by in silico and in vitro analyses. Therefore, the iron oxide based nanocarrier acts as an “iron shield”, protecting the highly reactive hydroxyl groups of EGCG. The advantage of this strategy was assayed in human cell cultures in which SAMN@EGCG displayed outstanding inhibitory activity on endogenous protein kinase CK2 comparable to that obtained with CX-4945, the most potent and selective CK2 inhibitor, thus substantiating the superiority of the as-obtained nanohybrid in comparison to free soluble EGCG.

Finally, we assessed the antimicrobial potential of SAMN@EGCG complex on *Pseudomonas aeruginosa*, the most important pathogen involved in cystic fibrosis (CF) [13]. CF is a genetic disorder characterized by progressive lung disease with thick mucus formation, chronic inflammation, and persistent and untreatable bacterial colonization and infections, which are of concern in public healthcare due to the high antibiotic resistance shown by bacterial strains isolated in CF patients. Interestingly, several in vitro studies showed the potential activity of EGCG against bacteria [14]. In this respect, our results demonstrated that the SAMN@EGCG nanohybrid is much more effective than the free soluble EGCG in one of the bacteria strains tested, resulting in a lowered minimum inhibitory concentration and thus confirming the general advantage of nano-immobilization of the active compound.

## 2. Materials and Methods

### 2.1. Materials

(−)-Epigallocatechin-3-gallate (cod. E4143) was purchased from Sigma-Aldrich (Milan, Italy). Iron oxide magnetic nanoparticles, named Surface Active Maghemite Nanoparticles (SAMNs), were obtained as described in [15]. Briefly, FeCl_3_·6H_2_O was dissolved in milli-Q grade water under vigorous stirring at room temperature. NaBH_4_ solution in ammonia (3.5%) was added to the mixture. After the reduction reaction occurred, the temperature was increased to 100 °C and was kept constant for 2 h under stirring. Then, the material was cooled again at room temperature and aged in water for 12 h. The final product, a red-brown powder that responds to an external magnet, was achieved, after several washes in water, by incubation at 400 °C for 2 h.

### 2.2. EGCG Binding on SAMNs

A stable colloidal stock suspension of SAMNs was obtained in milli-Q grade water upon sonication for 20 min at 50 KHz, 100 W, in an LBS1 ultrasonic bath (Falc Instruments, Treviglio, Italy), with a final concentration of 0.5 g L^−1^. The stock suspension of SAMNs was firstly autoclaved to remove all potential biological contaminants that could interfere with the biological analysis. Thereafter, all the procedures involving biological samples were conducted in sterile conditions. A series of Nd–Fe–B magnets (N35, 263–287 kJ/m^3^ BH, 1170–1210 mT flux density by Powermagnet, Gottmadingen, Germany) were used for the magnetic separation of SAMNs from the suspension media. In total, 3 washing steps were performed with a sterile solution of tetramethylammonium hydroxide (TMAOH) 50 mM, pH 7.4, for 10 min under constant agitation to buffer exchange before the binding procedure. After the washing steps, EGCG 100 µM was added to the SAMNs suspension (0.5 g L^−1^) and incubated for 1 h at room temperature in continuous agitation to promote the process of surface functionalization. At the end of the incubation period, the external magnet was applied to separate the as-obtained SAMN@EGCG nanohybrid from the supernatant. The supernatant was collected, and 2 further washes were performed for 20 min in agitation with sterile TMAOH 50 mM, pH 7.4, to remove the weakly interacting excess of EGCG. Finally, the SAMN@EGCG nanohybrid was suspended in sterile milli-Q water at the final concentration of 0.5 g L^−1^. The amount of bound EGCG was calculated by UV-Vis measurements from the disappearance of the absorbance peak at 277 nm in the supernatants (ε_277_ = 1.15 × 10^4^ M^–1^ cm^–1^), as described previously for different cargos [11,12].

### 2.3. Biophysical Characterization of the Nanohybrid

#### 2.3.1. UV-Vis Spectroscopy

Optical spectroscopy measurements were acquired with Cary 60 spectrophotometer (Varian Inc., Palo Alto, CA, USA) in quartz cuvettes (path length, 1 cm). Two types of analysis were performed. The first was aimed at the characterization of the changes of bare SAMNs optical properties upon EGCG binding. For this purpose, the spectra of a 100 µg mL^−1^ suspension of either bare SAMNs or SAMN@EGCG in TMAOH 50 mM, pH 7.4, were registered. The second analysis was aimed at the assessment of EGCG-binding capacity on the surface of SAMNs. The binding behavior was tested at a constant concentration of the organic compound (100 µM) and at 4 different concentrations of SAMNs (2.0, 1.0, 0.5, 0.2, g L^−1^). After the incubation step (see above), supernatant was recovered. The amount of bounded EGCG was determined from the reduction of EGCG absorbance peak at 277 nm in the supernatants. EGCG (100 µM in TMAOH 50 mM, pH 7.4) was used as a reference.

#### 2.3.2. Transmission Electron Microscopy

Transmission electron microscopy (TEM) images were acquired by a JEOL JEM-2010 microscope (JEOL Ltd., Tokyo, Japan) operating at 200 kV with a point-to-point resolution of 1.9 Å. Before measurements, samples were dispersed in ethanol and the suspension was treated by ultrasound for 10 min. A drop of dilute suspension was placed on a carbon-coated copper grid and allowed to dry by evaporation at room temperature. Samples in solution were placed onto a holey carbon film supported on a copper-mesh TEM grid (SPI Supplies, West Chester, PA, USA) and air-dried at room temperature.

#### 2.3.3. Nanohybrid Size and Charge

Nanoparticles and hybrids dispersions were characterized in terms of hydrodynamic size and size distribution as well as zeta potential (ζ) by dynamic light scattering (DLS) using the Zetasizer Nanoparticle Analyzer mod. ZEN3600 (Malvern Instruments, Malvern, UK) at T = 298 K as described previously [11]. Origin software with a log-normal function was used to perform the statistical analysis of the size distribution.

#### 2.3.4. FT-Infrared Spectroscopy

Fourier-transformed infrared (FT-IR) spectra were registered using IRAffinty-1S spectrometer (Shimadzu Corp., Kyoto, Japan) equipped with a diamond ATR analyzer and LabSolutions IR software (Shimadzu Corp., Kyoto, Japan), as described in [11].

### 2.4. Antioxidant/Antiradical Activity Determination

Equal amounts in terms of either EGCG (free soluble compound vs SAMN@EGCG) or of nanoparticle concentrations (bare SAMNs vs SAMN@EGCG) were assayed with 3 different antioxidant/antiradical activity methods. The DPPH^•^ scavenging activity was determined according to an assay modified from [16]. The decrease in absorbance at 515 nm of the stable 2,2-diphenyl-l-picrylhydrazyl radical after reaction with free and immobilized EGCG was measured at 0 min, 1 min, and every 10 min until reaching a plateau. The direct production of 2,2′-azinobis-(3-ethylbenzothiazoline-6-sulfonic acid) into a stable ABTS^•+^ radical was achieved through the reaction between ABTS and potassium persulfate. The absorbance reduction at 734 nm of the stable radical incubated with free and immobilized EGCG was read at 0 min, after 1 min, and every 10 min until reaching a plateau following the protocol, as indicated in [17]. The ferric ion-reducing antioxidant power (FRAP) leveraged the reducing activity of free and immobilized EGCG that was incubated for 30 min at 37 °C at low pH, during which the ferric-tripyridyltriazine (Fe^3+^-TPTZ) complex was reduced to a ferrous (Fe^2+^) form that had an absorption maximum at 593 nm. Different samples were analyzed as described in [18].

### 2.5. Cell Cultures and Treatments

Human ovarian cancer cells (HeLa) and embryonic kidney cells (293T) were grown in Dulbecco’s modified Eagle’s medium (DMEM) high glucose (4.5 g L^−1^), supplemented with 10% (*w*/*v*) fetal bovine serum, 2 mM L-glutamine, 100 U mL^−1^ penicillin, and 100 mg mL^−1^ streptomycin (Sigma, Darmstadt, Germany). Cells were cultured in humidified 5% (*v*/*v*) CO_2_ atmosphere at 37 °C. Cells were seeded (12 × 10^4^ cells/well) 24 h before the indicated treatments on 6-well plates in the same culture medium. After each treatment, cells were washed twice with ice-cold phosphate-buffered saline (PBS), scraped and lysed in a buffer containing Tris-HCl 50 mM pH 7.5, NaCl 150 mM, and NP40 1% (*v*/*v*). cOmplete™ protease (Roche, Basel, Switzerland) and phosphatase (Sigma Aldrich, Milan, Italy) inhibitor cocktails were added to the lysis buffer. Protein concentrations were determined by the Bradford method. Protein kinase CK2 activity was evaluated by assessing the phosphorylation level of Akt phospho-Ser129 using a specific monoclonal antibody (Abcam, Cambridge, UK), as previously described [11]. Equal amounts of proteins from each sample were separated on 11% SDS-PAGE, blotted on Immobilon-P membranes (Millipore, Darmstadt, Germany), and processed by Western blot analysis with the indicated antibodies. Chemiluminescence signals of the HRP-conjugated secondary antibodies were obtained using the iBright image station (Thermo Fisher Scientific, Waltham, MA, USA) and quantified by ImageJ software. Loading control was provided by anti-β-actin antibody.

### 2.6. Cell Viability Assay

Cell viability was detected by the common 3-(4,5-dimethylthiazol-2-yl)-3,5-diphenyltriazolium bromide (MTT) assay. Briefly, 10 × 10^3^ cells/well were seeded in a 96-well plate and incubated with the indicated conditions for 24 h. Then, 1 h before the end of the incubation, 10 µL of MTT solution (5 mg mL^−1^ in PBS) were added to each well. Incubations were stopped by adding 20 µL of lysis solution at pH 4.7. Plates were read for OD at λ = 590 nm in a Victor™ X4 2030 multilabel plate reader (Perkin Elmer, Waltham, MA, USA).

### 2.7. In-Silico Analysis

The human protein kinase CK2 α-subunit crystal structure was retrieved from the Protein Data Bank (PDB code: 6HOP). The structure was considered with a constitutive water molecule, while all the other ligands and cofactors were removed. The Molecular Operating Environment (MOE) 2020.09 (Chemical Computing Group ULC, Canada) was used to prepare protein by adding hydrogen atoms following standard geometries. To minimize contacts between hydrogens, the structure was subjected to Amber99 force field minimization until the root mean square deviation of conjugate gradient was <0.1 kcal · mol^−1^ ·Å^−1^. Protein charges were added using Protonate 3D tool (MOE). EGCG was prepared, minimized, and charged using MMFF94× force field. Parameters for Fe(III) were retrieved exploiting a 12-6-4 Lennard-Jones (LJ)-type nonbonded model [19], integrated in the AMBER force field [20] and validated as in [21]. Molecular dynamics (MD) simulations of a system containing 100 hydrated Fe(III) and 50 EGCG were performed with ACEMD [22] for 50 ns after an equilibration phase of 1 ns (positional restraints were applied on carbon atoms to solvent equilibration). MD simulations exploited the NPT ensemble (isothermal, isobaric) at constant pressure (1 atm, Berendsen method) and temperature (300 K, Langevin thermostat), with explicit water molecules (TIP3P model) as described in [23]. Docking experiments were performed using the MOE-Dock program; the most suitable docking parameters were chosen after a calibration phase exploiting a small database of known crystallized CK2 3D structures [24]. Triangle Matcher was exploited as the placement method, with GBVI/WSA dG as the docking scoring function, as in [11]. Molecular dynamics simulations and molecular docking were performed using Accelera Computing system MC4-Node and Alienware Computing system 17-R2.

### 2.8. Microbiological Analysis

The *Pseudomonas aeruginosa* ATCC^®^ 27853™ strain was purchased by American Type Culture Collection (ATCC) and stored at −80 °C; the PAO1 strain was generously provided by Federica Briani (University of Milan, Italy). Reactivation of *P. aeruginosa* strains was achieved in petri dishes with Mueller–Hinton agar medium by the 4-quadrant streaking method. Petri dishes were incubated at 37 °C for 24 h. A single colony of each strain was collected and was grown in 10 mL Mueller–Hinton medium (MH) for 18 h at 37 °C under constant agitation in order to obtain a pre-standardize inoculum. After the overnight culture, serial dilutions were performed starting from a test tube containing ~10^8^ CFU mL^−1^ to reach a working concentration of 10^4^ CFU mL^−1^. Cells concentration was monitored by plate count on MH agar (MHA) and by the optical density at 600 nm (OD_600_) using a Spectrophotometer Multiskan GO Microplate Readers (Thermo Fisher Scientific, Waltham, MA, USA). A minimum inhibitory concentration (MIC) assay was used to identify the lowest concentration of antimicrobial agents that inhibited the growth of the microorganism. To determine the *P. aeruginosa* MIC of EGCG and SAMN@EGCG, Nunc™ MicroWell™ 96-well microplates (Thermo Fisher Scientific, Waltham, MA, USA) were prepared in sterile conditions. Different concentrations of free soluble EGCG and either SAMN@EGCG nanohybrid or bare SAMNs (negative control) were tested. Appropriate positive and negative controls were added to each microplate replicate in order to avoid any possible factors affecting the result. Three different replicates were performed for each plate and all experiments were performed in duplicate. Multiskan Go Microplate Reader was used to continuously measure the optical density at λ = 600 nm of each sample at intervals of 5 min for 24 h at 37 °C. Blank controls of each dilution were applied to evaluate changes in absorbance and the interaction of broth with the EGCG and SAMN@EGCG. This system allowed the evaluation of the proper baseline for the growth curve evaluation. The lowest concentration that produced a significant inhibition (≥50%) of the growth of *P. aeruginosa* strains in comparison with the control was identified as the MIC. In order to confirm the MIC OD_600_, 100 µL of the 24 h broth cultures of each test were plated on MHA to define the log reduction with respect to the concentration of the inoculum. The results were reported as Δlog_10_ CFU mL^−1^ of the initial inoculum of the 24 h cultures. The minimum bactericidal concentration (MBC), defined as the lowest concentration of the antimicrobial agent that causes 99.9% reduction of viable cells in the initial inoculum, was determined after the treatment with either free soluble EGCG, bare SAMNs, or SAMN@EGCG. For this purpose, 100 µL recovered from the microwells where no growth was observed during MIC tests were sub-cultured in 5 mL of MH medium and incubated at 37 °C for 24 h. MBC was defined as the lowest concentration that did not show microbial growth on the 24 h sub-cultures. The definition of EGCG as a bacteriostatic or bactericidal agent was established according to MBC/MIC ratio, as described in [25].

### 2.9. Statistical Analysis

Statistical analysis was performed with GraphPad Prism software v.5, 2007 (GraphPad Software, San Diego, CA, USA). All values are expressed as means ± standard deviation (SD) of at least 3 replicates. A comparison of more than 2 groups was made with ANOVA using Bonferroni’s post-test. Differences were considered statistically significant at values of *p* < 0.05. A non-parametric combination test (NPC) was conducted with the free software NPC Test R10 using the Tippet combining function [26]. The partial and the global *p* values were determined for microbial growth features (Δlog_10_ CFU and % of OD_600_). Differences at *p* < 0.05 were considered statistically significant. Several convenient characters suggested the use of NPC: it is robust when the number of variables is larger than the number of observations; it is effective when data are not normally distributed; categorical variables can be used to define stratification; and finally, it does not require data transformation.

## 3. Results

### 3.1. EGCG Immobilization on the SAMN Nanoparticle Surface

**Figure 1:**
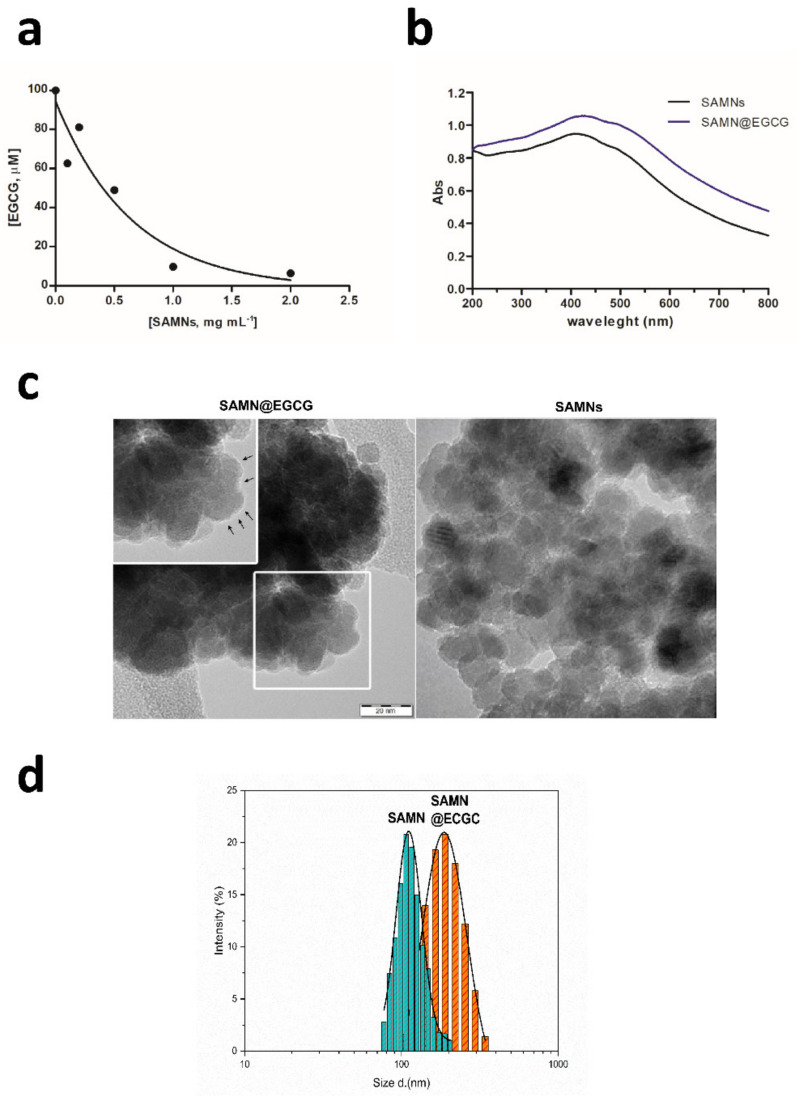
SAMN@EGCG nanocarrier characterization. (**a**) Bound EGCG on nanoparticles as a function of SAMN concentration. Measurements were carried out by spectrophotometry at 277 nm in water. (**b**) Optical spectra of naked SAMNs (black line) and of the SAMN@EGCG hybrid (blue line). (**c**) Representative HR-TEM micrographs of both the SAMN@EGCG hybrid (**right**) and bare SAMNs (**left**). In the inset, magnification of the selected area (white square). Arrows indicate organic shell due to immobilized EGCG. (**d**) Dynamic light scattering analysis of both bare SAMNs and the SAMN@EGCA complex. Measurements are expressed as a fractional distribution (%) versus hydrodynamic size (*d*, nm). The bold line represents the statistical-fit (LogNorm-function); measurements were carried out in triplicate.

Nanotechnology has been shown to create innovative products with new or improved functional properties. Among nanomaterials, iron oxide-based nanoparticles have been developed for diverse therapeutic or diagnostic purposes [27]. Recently, we demonstrated that surface active maghemite nanoparticles (SAMNs), with their unusual colloidal stability in water, high average magnetic moment, and unique spectroscopic and intrinsic photoluminescence properties, represent real competitors for standard technologies in drug delivery [11]. SAMNs form self-assembled core-shell nanohybrids by binding on surface chemical entities capable of participating in chelation processes. Given the chemical structure of (−)-Epigallocatechin-3-gallate (EGCG) and its complexing behavior with metal ions [28], the surface of bare SAMNs was promptly functionalized by simple incubation of 100 μM EGCG with a suspension of 0.5 g L^−1^ nanoparticles in 50 mM tetramethylammonium perchlorate, pH 7.4. After 1 h incubation at room temperature in continuous agitation, the as-obtained nanohybrid (SAMN@EGCG) was separated magnetically from the medium and washed extensively. The amount of immobilized EGCG was calculated by the disappearance of the specific absorbance peak at 277 nm (ε_277_ = 1.15 × 10^4^ M^−1^ cm^−1^) and resulted in 50 mg EGCG per gram of SAMNs (Figure 1a). All the operations were conducted in sterile conditions to allow the further use of the as-prepared nanohybrid for biological assays.

Usually, the binding of organic ligands on SAMNs surface changes the optical properties of the nanoparticles [11,12]. Indeed, UV-Vis spectra of SAMN@EGCG complex acquired in aqueous solution displayed a red-shift of both the wide band around 400 nm, with the peak shifting from 410 to 425 nm and the shoulder moving from 480 to about 500 nm (Figure 1b, compare blue vs black lines). This phenomenon has already been observed upon the binding of other biomolecules on SAMNs surface [29]. Interestingly, the optical red-shift of the maximum absorbance peaks is considered to be induced by surface modification of nanocrystalline metal oxide particles upon ligand binding [30].

Further, to corroborate the binding of the polyphenol molecule on the nanoparticle surface, the morphology of SAMN@EGCG nano-complex was analyzed by high-resolution transmission electron microscopy (HR-TEM). The HR-TEM micrograph of the as-obtained nanohybrid was characterized by nanoparticle aggregates with a well-defined spherical shape and size ranging from 10 to 20 nm, with the presence of a less electron dense brighter shell covering the crystalline maghemite core evidencing the organic layer (Figure 1c).

The hydrodynamic diameters of both bare SAMNs and the SAMN@EGCG core-shell complex were measured by dynamic light scattering (DLS) technique. As reported in Figure 1d, a substantial increase of the average hydrodynamic size from 115.20 nm ± 1.45 nm (*R*^2^ = 0.993) to 208.4 nm ± 1.7 nm (*R*^2^ = 0.998) was observed upon EGCG immobilization, demonstrating the presence of adsorbed ligand molecules on the surface of iron oxide nanoparticles. Interestingly, the polydispersity index, which represents the width of particle size distribution, for both bare SAMNs and the SAMN@EGCG complex was lower than 0.1. This result substantiated the monodispersity of SAMN@EGCG nanohybrid.

Bare SAMNs display a peculiar high positive zeta potential (ζ) equal to +31.8 ± 6.75 mV (conductivity 0.1151 mS cm^−1^ in water at 22 °C), that accords their high colloidal stability in solution without any surface modification or coating derivatization. Of note, upon adsorption of EGCG on the surface of SAMNs, ζ shifted to -31.6 ± 8.15 mV (conductivity 0.01151 mS cm^−1^ in water at 22 °C). Therefore, in accordance with the colloidal stability scale described in [31], the EGCG@SAMN nanohybrid can be considered as highly stable in solution.

**Figure 2:**
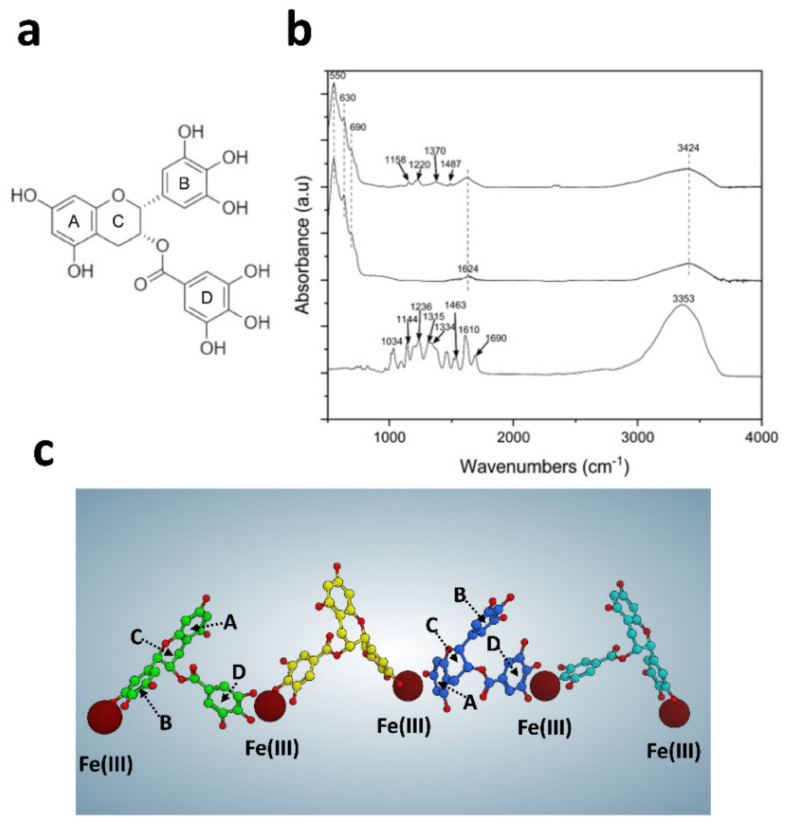
EGCG interaction with the SAMN surface. (**a**) Molecular structure of EGCG. (**b**) FT-IR spectra of bare SAMNs, the SAMN@EGCG nanohybrid, and EGCG from top to bottom, respectively. (**c**) Exemplary representation of iron(III) EGCG complexes obtained from MD simulation. The coordination between iron(III) and EGCG B- and D- rings (green, yellow, cyan), as well as EGCG A- and D- rings (blue), is shown.

As mentioned above, chemical entities possessing chelating moieties are able to bind directly to and functionalize the SAMN surface. EGCG, exposing eight phenolic hydroxyls, represents a versatile complexing agent able to chelate metal ions at each side of its molecule (Figure 2a). More specifically, it has been reported that EGCG forms a 2:1 metal/ligand complex with Fe(III), mainly through hydroxyls groups in the gallate ring (D-ring), and in the gallocatechin ring (B-ring) as a secondary coordination site [28]. A deeper and more direct insight into the molecular features involved in the chemical bonding process between SAMN nanoparticles and EGCG was obtained using Fourier-transform infrared (FT-IR) spectroscopy (Figure 2b). The FT-IR spectrum of SAMN@EGCG was compared to that obtained with free EGCG and bare SAMNs, respectively. EGCG exhibits a characteristic broad peak at 3353 cm^−1^ which corresponds to the stretching vibration of the eight hydroxyl groups decorating the aromatic rings [32,33]. In addition, EGCG presents other characteristic bands with medium intensity, as already reported in the literature. Specifically, the peak at 1690 cm^−1^ is attributable to the stretching of the carbonyl (C=O) group in the ester bond between the catechin scaffold and the galloyl moiety, whereas the peak at 1610 cm^−1^ refers to the aromatic C=C [34]. Moreover, EGCG shows a weak band at 1463 cm^−1^ for the presence of C-H alkanes in the chromane moiety [35]. The peaks at 1334 cm^−1^ and 1315 cm^−1^ are attributable to the alcohol C–O. Finally, the observed peaks at 1236 cm^−1^, 1144 cm^−1^, and 1034 cm^−1^ correspond to the deformation vibration of -OH groups in the aromatic rings [36], stretching of C-OH alcohols [35], and stretching vibration of the C-O-C group linked to the chroman and B- and D-rings [37], respectively.

As a control, bare SAMNs displayed strong bands in the low-frequency region ranging from 550 cm^−1^ to 690 cm^−1^, due to the vibrational and torsional modes of the binding between iron and oxygen atoms of the maghemite core [38]. The absorption bands near 1624 cm^−1^ and 3424 cm^−1^ are attributable to –OH bending and stretching vibrations derived from water-coordinating bare SAMNs, respectively [39,40].

As expected, the FT-IR spectrum of the SAMN@EGCG complex exhibited the characteristic peaks of bare SAMNs in the range between 690 cm^−1^ and 550 cm^−1^, as well as broad peaks at 3424 cm^−1^ and 1624 cm^−1^, due to the presence of the iron oxide core. Most importantly, in comparison to unmodified nanoparticles, the spectrum of the self-assembled complex presented additional vibrations in the range between 1100 and 1550 cm^−1^ that very likely can be ascribed to the presence of the immobilized organic molecules. Interestingly, after complexation the broad peak associated to the hydroxyl groups stretching vibration (3353 cm^−1^) decreased dramatically and the aromatic O−H as well as C-OH blue shifted and nearly disappeared from the FT-IR spectrum, respectively. This result, in agreement with the chelation mechanism of iron by EGCG [28], clearly indicates that during functionalization, the structure of EGCG changed and the hydroxyl groups strongly combined with undercoordinated Fe(III) sites on the surface of SAMNs. In addition, the disappearance of EGCG peak at 1690 cm^−1^ could be explained by the involvement of the carbonyl group in the ester bond in the formation of the SAMN@EGCG complex. The disappearance of EGCG peak at 1610 cm^−1^, attributed to aromatic C=C, can be interpreted in terms of an increase of molecular rigidity upon anchoring on the SAMN surface. Overall, such changes in the characteristic bands of free EGCG upon binding on SAMNs surface suggest that the Fe(III) chelation process is not restricted to the hydroxyl groups in the B- and D-rings [28]. Therefore, in order to provide an interpretation on EGCG binding orientation in the interaction between EGCG and Fe(III), a series of in silico studies were carried out, assuming that solvent-exposed Fe(III) sites on the SAMN surface behaved as Fe(III) ions in solution [23]. In particular, a system was created consisting of 100 Fe(III) interacting with 50 EGCG molecules. After an initial minimization step, the system was subjected to a molecular dynamics (MD) simulation protocol in explicit water. The MD results revealed that EGCG coordinates with Fe(III) in the ratio of 1:2 with a preferential binding site at the B- and D-rings (78%, Figure 2c). However, it was possible to identify a series of complexes where EGCG interacts with two iron molecules by exploiting the hydroxyls present in A-ring and B- or D-rings (9% and 13% respectively, Figure 2c). These results are in agreement with the data reported in literature [28] and with the FT-IR analysis, with particular reference to the significant decrease of the hydroxyl groups stretching vibration.

### 3.2. Nano-Immobilization Protects EGCG from Degradation

Catechins, acting as donors of hydrogen atoms or single electrons, are able to neutralize the damaging effects of reactive oxygen species (ROS), and EGCG is considered one of the most effective, promptly reacting with the majority of ROS [41]. Indeed, the presence of trihydroxy B-ring as well as of the gallate group in the 3-position of chromane moiety contribute mainly to electron delocalization and thus to free radical scavenging ability of EGCG. However, under physiological conditions and in cell culture media [8], such chemical features make EGCG unstable in solution due to rapid auto-oxidative degradation. This non-enzymatic reaction involves the dehydrogenation of hydroxyl groups in the B-ring, generating o-quinone that in turn dimerizes, leading to the formation of theasinensin A and compound P2. In addition, metabolic transformations (i.e.,methylation, glucuronidation, and sulfation) further affect the in vivo efficacy of EGCG [7]. For these reasons, several structural modifications have been proposed to increase the chemical/metabolic stability of EGCG by masking the hydroxyl groups [42]. FT-IR and MD data suggested that in SAMN@EGCG nanohybrid these functional groups participate in the binding with the nanoparticle surface. This implies that they might be unavailable to exert their scavenging activity, thus being in some extent preserved from auto-oxidative degradation.

**Figure 3:**
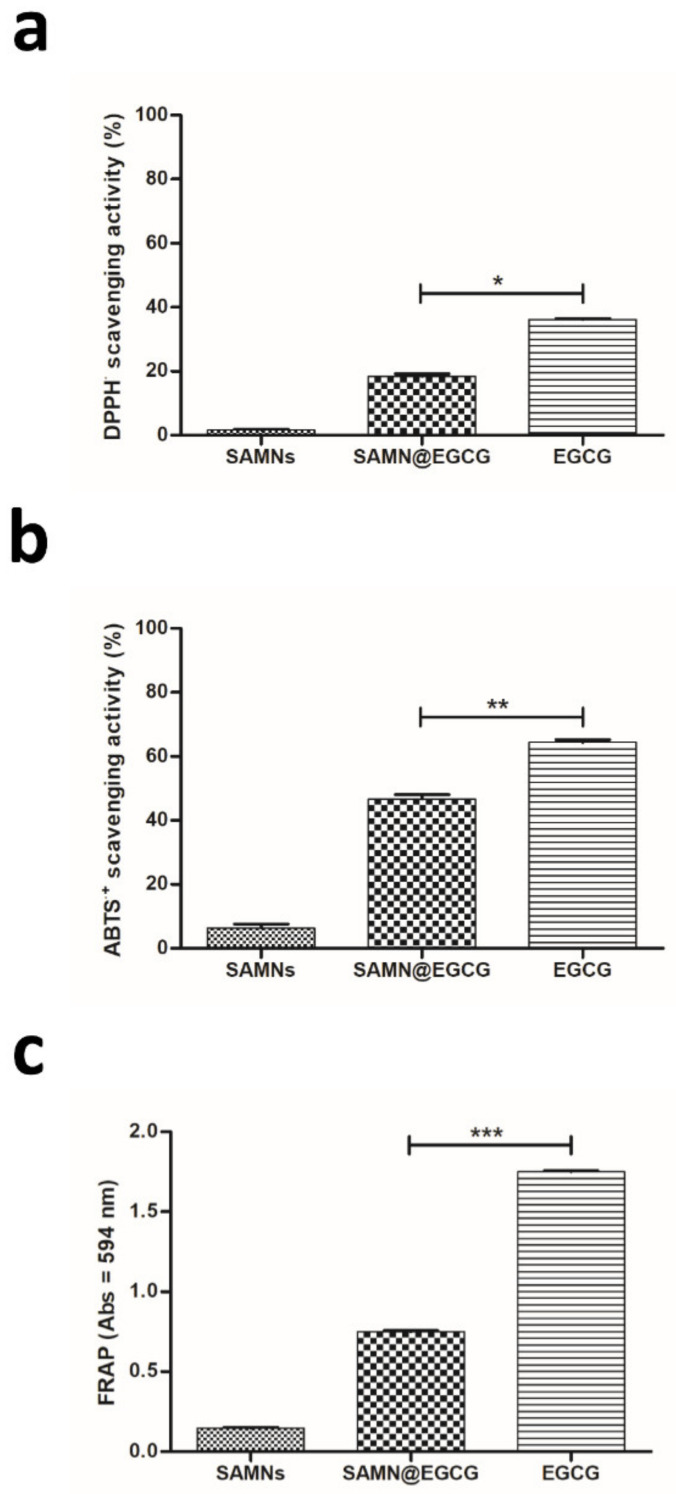
Antioxidant activity of SAMN@EGCG. (**a**) DPPH^•^ and (**b**) ABTS^•+^ radical scavenging activities of free soluble EGCG and immobilized EGCG. Bare SAMNs were used as negative control. The activity is presented as the percentage of scavenged free radicals initially added to the solution after 60 min of incubation with the indicated compounds. (**c**) Absorbance at 593 nm resulted from the formation of (Fe^2+^–TPTZ) complex after incubation with the indicated compounds. The same amounts of bound and soluble EGCG were assayed. The means ± SD of 3 independent experiments are reported; * *p* < 0.05, ** *p* < 0.01 and *** *p* < 0.001 compared to soluble EGCG (2-way ANOVA using Bonferroni’s post-test).

Therefore, to assess if nano-immobilization effectively protect the hydroxyl groups, we firstly evaluated the radical scavenging ability of the SAMN@EGCG nanohybrid. Equal concentrations of bound and soluble EGCG were assayed using DPPH^•^ and ABTS^•+^ methods (Figure 3a,b). Both methods rely on hydrogen exchange mechanism. While partially preserved, SAMN@EGCG displayed a reduced scavenging ability with respect to free soluble EGCG. This result is in accordance with the in-silico analysis that sampled two different populations of EGCG binding modes on the nanoparticle surface, leaving free the hydroxyl groups of either B-ring or D-ring, alternatively. Notably, the scavenging efficacy of SAMN@EGCG measured with the two methods varied considerably. This result reflects the different sensitivity of the two methods. Indeed, the ABTS^•+^ assay is strongly positively associated with oxygen radical absorbance capacity. Consequently, the free radical scavenging efficacy detected by ABTS^•+^ method is usually greater than that obtained with the DPPH^•^ test [43]. As a proof of such important variation, the different yields of radical scavenging activity obtained with equal amounts of free soluble EGCG (64% vs. 36% using ABTS^•+^ and DPPH^•^, respectively; see Figure 3a,b) should be noted. On the contrary, as depicted in Figure 3c, the antioxidant activity of immobilized EGCG measured by the ferric-reducing antioxidant power (FRAP) assay was strongly reduced (42.9% with respect to free EGCG). Note that the FRAP assay is a method based on the reduction of the colorless Fe^3+^-TPTZ complex to the intense blue Fe^2+^-TPTZ once it interacts with an antioxidant molecule that in turn is oxidized. Therefore, the impaired antioxidant effect measured for the nanohybrid by the redox-linked colorimetric reaction could be ascribed to the lack of resonance of the hydroxyl groups in meta and para due to their involvement in the chelation of Fe(III) on the nanoparticle surface, thus confirming that in our experimental conditions the nano-complex protects them from auto-oxidation process. Notably, bare nanoparticles did not interfere substantially with all the assays used.

### 3.3. SAMN@EGCG Enhances Cell Targeting of Endogenous CK2 in Human Cancer Cells

**Figure 4:**
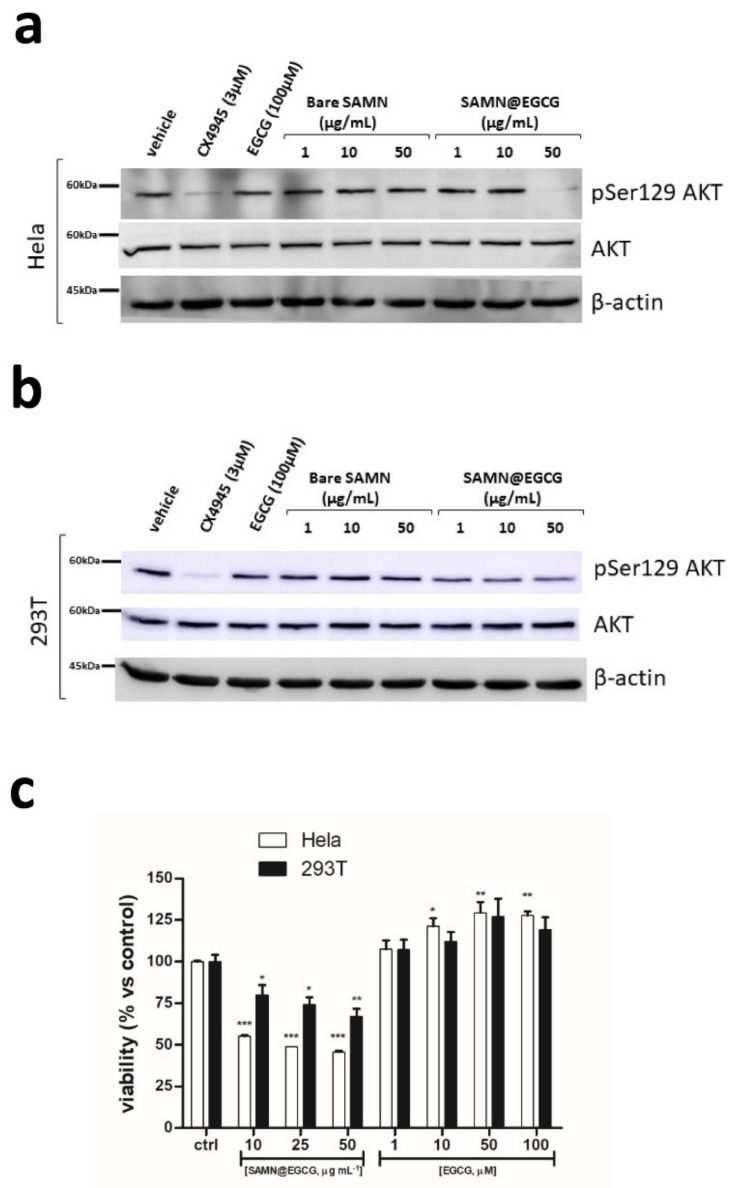
Enhanced cellular targeting and endogenous protein kinase CK2 inhibition by SAMN@EGCG nanocarriers in human cell cultures. Total cell lysates (15 μg of proteins/sample) from (**a**) Hela cells and (**b**) 293T cells treated for 16 h with the indicated treatments were immunoblotted with antibodies against both phospho-specific (S129) and total AKT. β-actin was used as loading control. Representative WBs of 3 independent experiments are shown. (**c**) Cytotoxicity evaluation of SAMN@EGCG nanohybrid. The viability of HeLa (white bars) and 293T (black bars) cells was measured by MTT assays after 24 h of treatment with the indicated concentrations of SAMN@EGCG and free soluble EGCG, respectively. The means ± SD of 3 independent experiments are reported; * *p* < 0.05, ** *p* < 0.01 and *** *p* < 0.001 compared to control (1-way ANOVA using Bonferroni’s post-test).

It has been suggested that the anti-cancer effects of EGCG are mediated through several mechanisms, such as the stimulation of antioxidant activity, the production of epigenetic changes, the generation of ROS that in turn inhibit cancer cell growth through the induction of apoptosis, and the impairment of different signaling pathways. Indeed, a plethora of biomolecules have been reported to interact directly with EGCG in cells [4]. The pro-survival protein kinase CK2, a pleiotropic, constitutively active Ser/Thr kinase, is frequently overexpressed in cancer [5]. Note that CK2 is not an oncogene, since its over-expression is generally not sufficient by itself to promote cancer development, but it boosts other oncogenic signals, making the environment more suitable for cancer progression, a phenomenon known as non-oncogene addiction [44]. For this reason, targeting CK2 represents an attractive option to counteract the growth and spread of different tumors, and several CK2 inhibitors have been developed so far [45]. Previously, we have demonstrated that in vitro EGCG is able to inhibit both the recombinant the CK2 holoenzyme and its isolated catalytic subunit with IC_50_ values in the nanomolar range [6]. To assess the advantage of using the as-prepared nanohybrid to enhance drug delivery of EGCG into target cells, Hela cells were treated for 16 h with increasing amounts of SAMN@EGCG (Figure 4a). Equal amounts of bare SAMNs were used as a negative control. The phosphorylation of Ser129 of AKT was monitored as a cellular marker of CK2 activity [11]. Of note, the CK2 inhibition obtained with 50 μg mL^−1^ of SAMN@EGCG, corresponding to 5.5 μM of free parent compound as calculated by the binding ratio (see above), was comparable to that obtained with 3 µM CX-4945, the most specific and potent ATP-directed CK2 inhibitor [45]. On the contrary, treatment with 100 μM free soluble EGCG, a concentration previously reported as being efficacious on prostate cancer cells [46], was completely ineffective in our experimental conditions. Similar results, with a less dramatic inhibition, were obtained in human embryonic kidney cells (Figure 4b).

To ascertain if the reduction of Ser129 phosphorylation level observed in cells treated with SAMN@EGCG corresponded to an enhanced efficacy of the nanohybrid in inducing tumor cell death, we performed cell viability experiments by the MTT method. Interestingly, as depicted in Figure 4c, SAMN@EGCG induced dose-dependent appreciable cell death in Hela cancer cells. Conversely, increasing concentrations of soluble EGCG did not affect cell viability; rather, they induced a slight proliferation. In 293T cells, the cytotoxicity induced by SAMN@EGCG was lower than that obtained in Hela cells. This result did not surprise and confirmed the lower sensitivity to CK2 inhibition displayed by a non-cancerous cell line [44].

**Figure 5:**
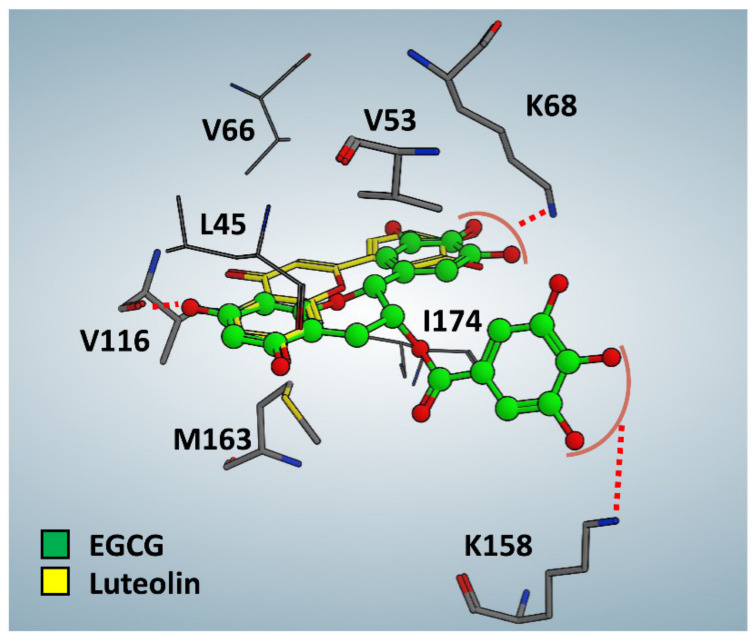
Docking analysis of EGCG in complex with human protein kinase CK2 α catalytic subunit. The best docking conformation of EGCG (green) compared to the crystallographic pose of luteolin (PDB code: 4DGN, yellow) is represented.

In a previous work, we showed that nano-immobilization of ferulic acid selectively directs the molecule on SAMNs into the CK2 kinase catalytic cleft without releasing it from the nanoconjugate [11]. In order to dissect the molecular interactions between immobilized EGCG and protein kinase CK2, we performed molecular docking studies of EGCG against human CK2. Considering that 3D structures of EGCG in complex with CK2 are not available, we used the crystallographic structure of the flavonoid luteolin in complex with *Zea mays* CK2 (PDB code: 4DGN) as a template. In fact, EGCG and luteolin exhibited several structural similarities. The best conformation obtained from the in-silico analysis showed that the EGCG conformation was stabilized by hydrophobic interactions with Leu 45, Val 53, Val 66, Phe 113, Val 116, Met 163, and Ile 174. On the other side, the hydroxyl group in position 7 (A-ring) was hydrogen bonded with the carbonyl group of Val 116 in the hinge region, while the hydroxyl groups of the B- and D-ring established a two-dipole ion interaction with Lys 68 and Lys 158, respectively (Figure 5). The in silico data, therefore, suggested that EGCG must necessarily be released from the nanoparticle surface in order to exploit CK2 inhibition, thus demonstrating that the increased CK2 inhibitory effect obtained with SAMN@EGCG was due to an efficient intracellular transport and delivery of EGCG by the nanocarrier.

### 3.4. EGCG Immobilization on Nanoparticle Surface Modulates Its Antimicrobial Potential against P. Aeruginosa

**Figure 6:**
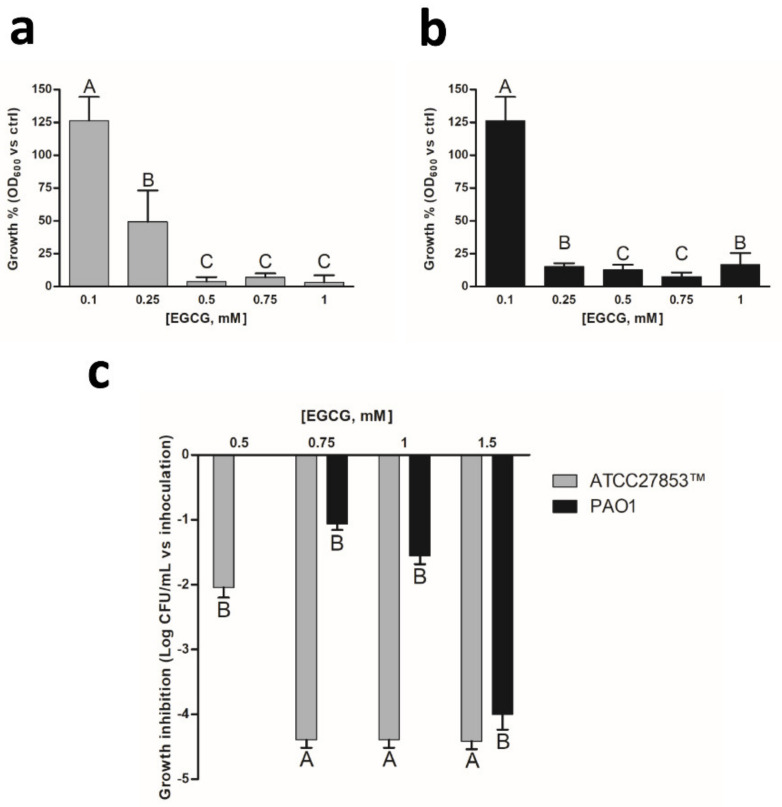
Inhibitory effect of free soluble EGCG on *P*. *aeruginosa* growth. Different concentrations of free soluble EGCG were assayed on *P*. *aeruginosa* (**a**) ATCC27853™ and (**b**) PAO1 strains. The growth percentages of each concentration with respect to the control are reported. (**c**) Delta growth inhibition after 24 h treatment with EGCG at the indicated concentrations. Different upper-case letters denote significant differences (*p* < 0.01) between treatments according to pairwise comparison among concentrations. Mean values ± SD are reported. Contrasts were performed for each strain independently.

Given the protective effect granted by nano-immobilization to EGCG, we wondered if the nanohybrid might also represent an advantage in the treatment of bacterial infections. In particular, among pathogens causing community- and hospital-acquired infections, *Pseudomonas aeruginosa* generates about 10% of all nosocomial infections in immunocompromised patients, being one of the major causes of mortality and morbidity in patients affected by cystic fibrosis [47]. It should be noted that the antibacterial activity of EGCG against *P. aeruginosa* has been reported previously, with minimum inhibitory concentrations (MICs) ranging from 200 to 800 µg mL^−1^ (i.e., 440–1740 µM). However, its efficacy for this class of Gram-negative bacteria remains somewhat controversial [48]. For this reason, we firstly assessed the antibiotic activity of soluble EGCG by performing quantitative assays of MICs against two distinct strains of *P. aeruginosa*, namely ATCC27853™ and PAO1. As depicted in Figure 6, soluble EGCG confirmed its ability to inhibit *P. aeruginosa* growth but at concentrations lower than those reported in literature [49]. Interestingly, after 24 h of treatment, PAO1 seemed more sensitive to EGCG (MIC = 250 µM) compared to ATCC27853™ (MIC = 500 µM) (Figure 6a,b). On the contrary, the measurement of the minimum bactericidal concentration (MBC) evidenced that EGCG possesses a higher potency against ATCC27853™ with respect to PAO1 (MBC 750 µM vs 1.5 mM, respectively). In particular, as reported in Figure 6c, concentrations ranging from 0.75 to 1.5 mM showed the reduction of more than 4 ΔLog CFU mL^−1^ for ATCC27853™, suggesting a strong bactericidal effect against this *Pseudomonas* strain. A similar reduction was obtained against PAO1, but only with 1.5 mM EGCG. Therefore, according to Levison et al. [25], EGCG should be considered as bactericidal for ATCC27853™ (MBC/MIC ≤ 4) and bacteriostatic for PAO1 (MBC/MIC > 4).

**Figure 7:**
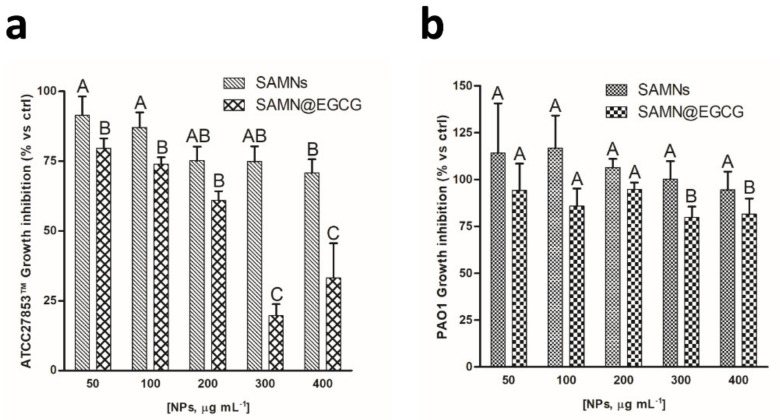
Inhibitory effect of SAMN@EGCG nanohybrid on *P*. *aeruginosa* growth. Different concentrations of SAMN@EGCG nanohybrid and bare nanoparticles were assayed on *P*. *aeruginosa* (**a**) ATCC27853™ and (**b**) PAO1 strains. The effect of each concentration on bacterial growth is expressed as percentages with respect to the control. Different lower-case letters represent significant (*p* < 0.01) differences between treatments according to pairwise comparison among concentrations. Mean values ± SDs are reported. Contrasts were performed for each strain independently considering SAMNs and SAMN@EGCG as separate treatments.

Thereafter, the antimicrobial efficacy of the SAMN@EGCG nonohybrid was studied in comparison to bare SAMNs in the concentration range between 50 and 400 µg mL^−1^. Notably, after 24 h of incubation, the MIC value shown by SAMN@EGCG on ATCC27853™ was 300 μg mL^−1^ (Figure 7a). Considering the amount of EGCG on SAMNs (50 mg g^−1^ of SAMNs), the MIC obtained with the nanohybrid resulted two orders of magnitude lower than that measured with soluble EGCG (32.7 μM vs 500 µM, *p* = 0.001), demonstrating the advantage given by EGCG nano-immobilization. However, the nanohybrid was not able to kill the bacteria completely, as evidenced by the impossibility with regard to the evaluation of the MBC. On the other hand, SAMN@EGCG was completely ineffective on the PAO1 strain and only a partial inhibitory effect was observed on bacteria growth at the highest concentrations tested (Figure 7b). Notably, bare SAMNs did not display any substantial effect on bacterial growth in both the strains tested.

## 4. Discussion

In the present work, remarkable iron oxide nanoparticles, which combine supermagnetism and intrinsic fluorescence with high colloidal stability [11,15], were used to develop a self-assembled core-shell nanocarrier of the bioactive polyphenol EGCG. The as-obtained SAMN@EGCG nanohybrid represents an interesting example for the study of how nano-immobilization can modulate and select the multifunctional properties of the EGCG molecule. Intriguingly, upon surface conjugation, EGCG was able to inhibit the endogenous protein kinase CK2 in the same concentration range as the most potent and specific CK2 inhibitor, CX-4945 (5.5 µM vs. 3 µM). This result is quite impressive since EGCG is not considered a selective CK2 inhibitor [6,50]. Further, our data showed that treatments with up to 100 µM of free soluble compound do not affect CK2 activity in cells, conversely to what has been reported in a different cell line [46]. Overall, these results highlight the importance of drug targeting in modern medicine. In fact, thanks to SAMN@EGCG nanohybrid it is possible to make active a compound, that, when separated from its “carrier”, would be almost ineffective. Further, our results demonstrate how nano-carrier development based on peculiar nanoparticles can support elements of pharmaceutical technology already known to efficiently target drugs, such as hydrogels or liposomes. Note that protein kinase CK2 takes part in some of the key pathways that control cell proliferation and survival in cancer progression, including the Wnt signaling, JAK/STAT, NF-κB, and PTEN/PI3K/Akt-PKB pathways [51]. Therefore, the efficient inhibition of CK2 obtained with SAMN@EGCG nanohybrid unveils a new important target of this natural compound that can be considered a common denominator for cancer therapy given its anti-apoptotic and pro-survival roles, among others, that in turn promote cancer progression. Most importantly, the greatly enhanced intracellular targeting displayed by the nanohybrid underlines the protective role granted by the surface conjugation. Indeed, free EGCG is not stable under most experimental conditions due to auto-oxidation and dimerization reactions [52] that negatively condition the performance of the compound as a therapeutic agent. Conversely, upon nano-immobilization, EGCG does not undergo rapid degradation since its highly reactive hydroxyl groups, being involved in the binding with the nanoparticle surface, are shielded by the iron oxide nanocarrier, as confirmed by the reduced radical scavenging ability of the nanohybrid. The protective effect combined with the efficient cell internalization of SAMNs leads to the efficacious delivery of the nano-immobilized EGCG inside the cell where, as suggested by in silico data, it is released from the nanoparticle surface and inhibits endogenous CK2.

In addition, we evaluated the antibacterial activity of both free soluble EGCG and SAMN@EGCG against Gram-negative *Pseudomonas aeruginosa* strains. Of note, treatment of infections caused by this pathogen is challenging due to the high prevalence of multidrug resistance against commonly used antibiotics, especially in immunocompromised patients. Interestingly, it has been suggested that the damage caused to bacteria by EGCG is promoted mainly by H_2_O_2_ production and oxidative stress induced by the phytochemical [53]. Moreover, it has been proposed that EGCG does not enter into bacterial cells, but rather that it exerts antibiotic effects at the cell membrane [14]. Several studies have demonstrated that EGCG actively inhibits the growth of Gram-positive bacteria. On the contrary, EGCG possesses a lower antibiotic efficacy against Gram-negative bacteria, displaying minimum inhibitory concentrations 8- to 16-fold higher than that obtained against Gram-positive bacteria [49,54]. This intrinsic resistance is ascribed to the protection given by the negatively charged lipopolysaccharides on the outer membrane of Gram-negative bacteria that hinder the electrostatic interactions with the polyphenol molecules [49]. Nevertheless, atomic force microscopy studies revealed that EGCG induces temporary instability of the *P. aeruginosa* cell membrane [53]. This effect could be accounted for by the direct binding of EGCG to specific surface membrane proteins expressed by Gram-negative bacteria. In this respect, in *Escherichia coli*, Nakayama et al. [55] demonstrated the intimate molecular interaction between EGCG and the outer membrane porin OmpG, resulting in the inhibition of its function by steric blocking of the pore access. Note that in the outer membrane of Gram-negative bacteria, several porins control the cellular uptake of small molecules such as nutrients and antibiotics, and the balance between the relatively limited porin-mediated passive uptake across the outer membrane and the active efflux in the inner membrane via efflux pumps creates a barrier for many antimicrobial agents [56].

If on one hand our results confirmed the antibiotic effect of free soluble EGCG on both the *P. aeruginosa* strains tested, the SAMN@EGCG nanohybrid was demonstrated to be much more effective than the soluble compound, at least on ATCC27853™ strain (MIC 32.7 μM vs. 500 μM). Considering the mechanisms proposed for EGCG antimicrobial activity, it is plausible to hypothesize that the enhanced antibiotic potency displayed by the nanohybrid might be due to the concentration of the active molecule on the outer layer of the bacteria. This effect could be promoted by the selective binding of SAMNs to OprF, an outer membrane protein of *Pseudomonas* spp. with a broad range of functions [57]. In fact, OprF was previously identified as the specific biological component involved in the contact between *P. fluorescence* and SAMNs [58]. Consistently, in previous work we demonstrated that bare SAMNs are not internalized by other Gram-negative bacteria [12]. Therefore, the proximity of the active compound to the bacteria surface conferred by the interactions between the nanocarrier and the outer membrane porin may enhance the oxidative stress induced by EGCG to *P. aeruginosa*. Nevertheless, the inhibitory effect displayed by the colloidal nanohybrid on the growth of ATCC27853™ strain was completely lost when tested on PAO1. This difference may be related to the different genomic and transcriptomic profiles of ATCC27853™ and PAO1 [59]. Indeed, the comparative analysis of the two strains revealed distinctive surface characteristics and detoxification systems that can support the different susceptibility observed with either soluble EGCG or the nanohybrid. In particular, the ineffectiveness of the nanohybrid against PAO1 growth may be explained by the relatively low amount of the active compound carried by nanoparticles that in turn could be insufficient to sustain antimicrobial activity on this bacterial strain. In addition, the lack of bactericidal effect exhibited by SAMN@EGCG on both *P. aeruginosa* strains, a phenomenon that we have previously described for a different nano-antibiotic [12], further supports this hypothesis.

## 5. Conclusions

In conclusion, our results demonstrate that SAMNs may represent a valuable nano-platform for drug carrier, effectively protecting the loading compound and enhancing its intracellular delivery and targeting. Indeed, nano-immobilization of EGCG onto SAMN surfaces turned it into a prospective therapeutic, with improved characteristics that can be further explored for alternative cancer treatments.
